# FcεRII (CD23) in dermatological disorders: structure, function, and clinical implications

**DOI:** 10.3389/falgy.2026.1849342

**Published:** 2026-08-10

**Authors:** Natalia Zdanowska, Wiktoria Nowik-Zając, Joanna Czerwińska, Joanna Najbar, Agnieszka Owczarczyk-Saczonek

**Affiliations:** 1 Katedra i Klinika Dermatologii, Chorób Przenoszonych Drogą Płciową i Immunologii Klinicznej, UWM; 2 Studenckie Koło Naukowe przy Katedrze Dermatologii, Chorób Przenoszonych Drogą Płciową i Immunologii Klinicznej, UWM

**Keywords:** allergy, atopic dermatitis, bullous pemphigoid, CD23, chronic urticaria, IgE

## Abstract

FcεRII (CD23) is the low-affinity receptor for IgE with unique structural and functional properties that position it as both a regulator and an effector in type I hypersensitivity. Unlike FcεRI, CD23 is a type II C-type lectin that exists as membrane trimers (CD23a/CD23b) and as soluble fragments (sCD23) generated mainly by ADAM10. Through context-dependent mechanisms, including co-ligation with CD21 on B cells, oligomerization-dependent IgE binding, and interactions with integrins, CD23 can either suppress or amplify IgE synthesis, shape antigen presentation, and modulate downstream immune responses. Importantly, accumulating experimental and clinical evidence indicates that CD23 exerts a predominantly regulatory role in IgE homeostasis, although its soluble forms may contribute to amplification of inflammatory signaling in specific contexts. Emerging dermatologic data link CD23 to atopic dermatitis, chronic spontaneous urticaria, and bullous pemphigoid, where circulating soluble CD23 often correlates with total IgE and disease activity. This narrative review is based on a structured PubMed search (1980–2024) using predefined keywords related to CD23, IgE, and dermatological diseases. We synthesize current knowledge on CD23 structure and biology, its physiological and immunoregulatory roles, and disease-specific evidence in dermatology. We also discuss translational implications, including modulation of CD23 cleavage and anti-IgE strategies. Finally, we highlight key gaps that must be addressed to validate CD23 as a biomarker and therapeutic target in skin disease.

## Introduction

Immunoglobulin E (IgE) is a central driver of type 2 inflammation and contributes to the pathobiology of several common dermatologic conditions, including atopic dermatitis, chronic spontaneous urticaria, and selected autoimmune blistering diseases such as bullous pemphigoid (3, 4, 9–12, 14). Beyond its well-known effector arm mediated by the high-affinity receptor FcεRI on mast cells and basophils, IgE biology is regulated by the low-affinity receptor FcεRII (CD23), which integrates control of IgE production with IgE-facilitated antigen handling (1, 2, 4, 6, 25). This regulatory layer is increasingly relevant to dermatology, as it links disease activity to measurable circulating markers and identifies additional therapeutic entry points within the IgE network.

CD23 is a type II transmembrane C-type lectin encoded by the *FCER2* gene (1, 2, 4). It is constitutively expressed on B cells and can be induced by interleukin-4 on a broader range of immune and epithelial cells (1, 2, 4, 25). Proteolytic shedding of membrane-bound CD23, primarily mediated by ADAM10, generates soluble CD23 fragments with cytokine-like properties (1, 2, 4, 5). Importantly, membrane-bound and soluble forms of CD23 exert divergent effects on IgE homeostasis. Membrane CD23 is generally associated with negative feedback regulation of IgE synthesis, whereas soluble CD23 may enhance IgE production and amplify downstream inflammatory signaling (1, 2, 4, 5, 24, 25). In dermatologic cohorts, circulating soluble CD23 has been reported to correlate with total IgE levels and, in some diseases, with clinical activity, supporting its evaluation as a candidate biomarker and a component of future stratification strategies.

Mechanistic studies further indicate that CD23 participates in IgE-facilitated antigen presentation and may shape epithelial–immune crosstalk (6). Structural analyses have demonstrated that IgE cannot bind FcεRI and CD23 simultaneously, as these receptors preferentially recognize distinct conformational states of the IgE Cε3 domain (3). This allosteric switch suggests a dynamic regulatory balance between effector activation and IgE homeostasis.

From a clinical perspective, the most consistent dermatology-focused data concern bullous pemphigoid, in which elevated serum and blister fluid levels of soluble CD23 have been associated with disease activity and IgE-related disease phenotypes (7–10, 36). In atopic dermatitis and chronic spontaneous urticaria, available studies suggest potential associations between soluble CD23, total IgE, and disease activity; however, findings are more heterogeneous, and comparisons across cohorts are limited by assay variability and inconsistent reporting (4, 11, 12, 21, 33–35). These limitations underscore the need for standardized methods for soluble CD23 measurement and harmonized clinical endpoints in future studies.

The aim of this narrative review is to synthesize current knowledge on the structure and biology of FcεRII (CD23), delineate the functional differences between its membrane-bound and soluble forms, and critically appraise evidence supporting its role in IgE-driven skin diseases, including atopic dermatitis, chronic spontaneous urticaria, and bullous pemphigoid. We further discuss translational implications of targeting the CD23–IgE axis and outline key research priorities required to validate soluble CD23 as a biomarker and to define its potential role in treat-to-target strategies in dermatology.

Importantly, the role of CD23 in allergic disease should not be interpreted as purely pro-inflammatory. While early studies emphasized its involvement in IgE-mediated responses, more recent experimental and translational data indicate that CD23 functions as a context-dependent regulator of IgE homeostasis (1, 2, 4, 24, 25). In particular, membrane-bound CD23 contributes to negative feedback control of IgE synthesis, whereas dysregulation of its cleavage and increased levels of soluble CD23 may shift this balance toward amplification of inflammatory pathways. This dual and dynamic role underscores the need for a more nuanced interpretation of CD23 as a modulatory rather than strictly pathogenic component of the IgE network.

## Structure and biology of FcεRII/CD23

FcεRII (CD23) is one of the two principal receptors for immunoglobulin E, in contrast to the high-affinity FcεRI. CD23 belongs to the C-type lectin family and exhibits low intrinsic affinity but high functional avidity for IgE through trimerization (1–4, 15).

The *FCER2* gene is located on chromosome 19p13.3 (1, 4). CD23 is a type II membrane protein composed of a short N-terminal cytoplasmic tail, a single transmembrane domain, a coiled-coil stalk region enabling dimer and trimer formation, and a C-terminal lectin domain responsible for IgE binding (1, 2, 4, 15). An RGD (arginine–glycine–aspartic acid) motif within the lectin domain mediates interactions with integrins, including αMβ2 and αXβ2 on myeloid cells and αvβ3/αvβ5 on epithelial cells, linking CD23 to leukocyte adhesion and barrier–immune communication (1, 4). Two isoforms have been identified: CD23a, constitutively expressed on B cells, and CD23b, inducible by interleukin-4, CD40 ligand, or Epstein–Barr virus infection, and expressed on a broader range of hematopoietic and non-hematopoietic cells (1, 2, 4, 17, 25).

Membrane-bound CD23 (mCD23) and its soluble fragments (sCD23) differ in origin, function, and clinical relevance. While membrane-bound CD23 is primarily involved in negative feedback regulation of IgE synthesis, soluble CD23 exerts cytokine-like activity and can amplify IgE responses (1, 2, 4, 5, 24, 25). A comparison of the biological properties of membrane-bound and soluble CD23 is provided in Table 1.

**Table 1:** Comparison of the functions of mCD23 vs. soluble CD23.

Feature	mCD23	sCD23
Form	Membrane-bound trimer (1, 4)	Soluble fragments (5)
Origin	Expressed on B cells, epithelial and myeloid cells (61)	Generated by ADAM10 or proteases (e.g., Der p1) (1)
IgE regulation	Suppresses IgE synthesis (negative feedback via CD21) (2, 4)	Enhances IgE synthesis (positive feedback via CD21) (2, 4)
Other functions	Facilitates antigen presentation, regulates B cell survival (1, 4)	Cytokine-like activity, induces NO and cytokine release (1)
Clinical significance	Potential therapeutic target (stabilization reduces IgE) (13)	Biomarker in BP, CSU, AD, malignancies (7–9)

For comparison, FcεRI, the high-affinity receptor for IgE, differs fundamentally from CD23 in structure, affinity, cellular distribution, and biological function. FcεRI is predominantly expressed on mast cells and basophils and serves as the principal trigger of degranulation and mediator release. The key structural and functional differences between FcεRI and FcεRII (CD23) are summarized in Table 2.

**Table 2:** Overview of the structural and functional differences between FcεRI and FcεRII.

Feature	FcεRI	FcεRII (CD23)
Structure	Tetramer (αβγ2) or trimer (αγ2) (14)	Type II C-type lectin trimer (15)
Affinity for IgE	High affinity (10^10^ M⁻^1^) (14)	Low affinity (∼10^8^ M⁻^1^) but high avidity via oligomerization (3, 15)
Expression	Mast cells, basophils, dendritic cells (Gołąb, 2021)	B cells, monocytes, eosinophils, epithelial cells (Gołąb, 2021)
Main functions	Effector cell activation, degranulation, mediator release (14)	Regulation of IgE synthesis, antigen presentation, immune modulation (1, 4)

Structural analyses have demonstrated that IgE cannot bind FcεRI and CD23 simultaneously. FcεRI preferentially recognizes the “open” conformation of the IgE Cε3 domain, whereas CD23 binds the “closed” Cε3 conformation, rendering these interactions mutually exclusive (3). This conformational selectivity establishes an allosteric switch between effector activation and regulatory control of IgE homeostasis.

In addition to its role in IgE binding, CD23 participates in active epithelial transport processes. CD23 mediates bidirectional transcytosis of IgE and IgE–allergen complexes across epithelial barriers, including the gastrointestinal and respiratory tract epithelia (4, 49). During this process, allergens are protected from intracellular degradation, allowing intact allergen delivery to subepithelial immune compartments where they can initiate or amplify immune responses (4). Functional studies using epithelial cell models demonstrated that silencing CD23 expression markedly reduces IgE transport in both apical-to-basolateral and basolateral-to-apical directions (49). Furthermore, engagement of epithelial CD23 by IgE–allergen complexes induces secretion of the chemokine CCL20, thereby promoting recruitment of immune cells and local inflammatory responses (4).

Engagement of FcεRI on mast cells and basophils promotes immediate effector responses, whereas membrane CD23 contributes to stabilization of negative feedback on IgE synthesis. In contrast, s soluble CD23 supports positive feedback mechanisms and cytokine-like signaling through interactions with CD21 and integrins.

CD23 expression is tightly regulated by cytokine signaling and environmental factors. Interleukin-4 and interleukin-13 upregulate CD23 expression, whereas interferon-γ suppresses it (1, 2, 4, 25). Proteolytic cleavage of CD23, mainly mediated by ADAM10 or environmental proteases such as Der p 1, results in the release of soluble CD23 fragments that retain IgE-binding capacity (16, 22).

Viral infections represent a well-documented mechanism of CD23 modulation. Epstein–Barr virus induces CD23 expression via EBNA-2–dependent transcriptional activation and is associated with B-cell transformation and proliferation (17). Similarly, Kaposi's sarcoma–associated herpesvirus replication and transcription activator (RTA) strongly induces CD23a expression through RBP-Jκ binding sites, leading to enhanced CD23 cleavage and release of soluble CD23 fragments (18). Increased CD23 expression has also been reported on peripheral blood monocytes in HIV-1 infection, reflecting an aberrantly activated monocyte phenotype (19), and on B lymphocytes in infants with respiratory syncytial virus infection, where it correlates with virus-specific IgE and IgG4 production (20).

Environmental and microbial factors further influence CD23 regulation. Although direct evidence linking gut or skin microbiota to CD23 expression is limited, indirect observations suggest modulation through cytokine signaling pathways. In atopic dermatitis, colonization with *Staphylococcus aureus* has been associated with increased CD23 expression on peripheral blood lymphocytes (22). Protease inhibitors such as alpha-1 antitrypsin suppress CD23 cleavage, whereas environmental pollutants, including tobacco smoke, inhibit this protective effect, potentially enhancing allergic susceptibility. Cockroach allergens and fungal pathogens can also modulate CD23 expression and function, contributing to innate and adaptive immune responses (23, 28).

Aberrant CD23 expression has diagnostic relevance in B-cell malignancies, including chronic lymphocytic leukemia, mantle cell lymphoma, and plasmacytoma. Elevated circulating soluble CD23 levels have also been reported in autoimmune diseases such as systemic lupus erythematosus, rheumatoid arthritis, and Sjögren's syndrome, suggesting broader utility as a biomarker of chronic immune activation (1, 45, 48).

Current evidence linking CD23 and soluble CD23 to dermatological disorders, together with their proposed clinical significance, is summarized in Table 3.

**Table 3:** Current evidence linking CD23 and its soluble form (sCD23) to various dermatological disorders.

Disease	sCD23 level	Clinical significance
Atopic dermatitis	Elevated; correlates with total IgE and activity; 314.5 ± 47.9 U/mL (mean ± SE; range: 203.6–455.1 U/mL), measured in serum by ELISA (CELLFREE T-Cell Diagnostic kit) (Hatzistilianou 1996; Engeroff 2021; Platzer 2011)	Potential biomarker of severity (Engeroff 2021)
Chronic spontaneous urticaria	Variable; may reflect endotype (IgEdriven vs. autoimmune) 16.6 IU/mL (median; IQR: 7.4–29.5 IU/ mL; controls: 22.1 IU/mL, IQR: 17.4– 27.6 IU/mL; serum, ELISA, Bender MedSystems) (Puccetti 2005; Kolkhir 2022; Brás 2023; Larenas-Linnemann 2023; Xiang 2025)	May stratify patients, linked to omalizumab response (Brás 2023; Larenas-Linnemann 2023)
Bullous pemphigoid	Elevated in serum and blister fluid; correlates with activity and eosinophilia 17.8 ± 6.5 µg/L (mean ± SEM; controls: 8.1 ± 1.5 µg/L; serum, sandwich ELISA using anti-CD23 mAbs BU-38/H107) (Furukawa 1994; Maekawa 1995; Messingham 2019; Seyed Jafari 2019)	Useful biomarker; supports IgE–CD23 axis in pathogenesis (Messingham 2019; Seyed Jafari 2019)
B-cell malignancies	Very high (esp. CLL); diagnostic/prognostic marker; 45– 8,656 U/mL (range; serum; IgEbinding factor/sCD23 assay) (Sarfati 1988; Acharya 2010)	Diagnostic marker; prognostic biomarker (Acharya 2010)
Autoimmune diseases (RA, SLE, SS)	Elevated; reflects chronic inflammation **RA:** 15.90 ± 4.20 ng/mL (mean ± SEM; controls: 4.23 ± 0.61 ng/mL; serum, immunoradiometric assay using antiCD23 mAbs) (Chomarat 1993); **SLE:** 18.1 arbitrary units (median; controls: 8.6 arbitrary units; serum, enhanced chemiluminescent sandwich ELISA) (Bansal 1992); **primary SS:** 23.0 arbitrary units (median; controls: 8.6 arbitrary units; serum, enhanced chemiluminescent sandwich ELISA) (Bansal 1992)	Proinflammatory mediator, potential biomarker (Acharya 2010)

Values are presented as reported in the original publications because sCD23 assays used different calibration standards and units; therefore, direct conversion between U/mL and mass concentrations was not performed.

## Physiological functions of FcεRII/CD23

### Preclinical evaluation of immunological pathways

CD23 functions as both a regulator and an effector within IgE biology. Its membrane-bound (mCD23) and soluble (sCD23) forms exert opposing effects, shaping immune responses in a context-dependent manner (1, 2, 4, 5).

#### Regulation of IgE synthesis

Membrane-bound CD23 suppresses IgE production by stabilizing surface IgE on B cells and engaging complement receptor 2 (CD21), thereby providing negative feedback on IgE synthesis (1, 2, 4, 24, 25). In contrast, trimeric soluble CD23 amplifies IgE production through co-ligation of CD21 and membrane-bound IgE on B cells (1, 2, 4, 5, 25). This bidirectional feedback mechanism allows CD23 to fine-tune IgE levels in response to antigen load and IgE availability. Experimental evidence consistently supports a regulatory role of CD23 in IgE homeostasis. CD23-deficient mice exhibit markedly elevated serum IgE levels, increased allergen-specific IgE and IgG1 production, and enhanced airway eosinophilia and hyperresponsiveness (24). In contrast, transgenic overexpression of CD23 suppresses IgE synthesis and limits the development of allergic inflammation. These findings indicate that CD23 functions as a negative regulator of allergic sensitization *in vivo* (36).

The regulatory effects of CD23 depend not only on its membrane localization but also on the structural characteristics of its soluble forms. Membrane-bound CD23 inhibits ongoing IgE synthesis through negative feedback mechanisms, whereas soluble CD23 can exert either inhibitory or stimulatory effects depending on its oligomeric state. Monomeric soluble CD23 tends to suppress IgE production, whereas oligomeric forms promote B-cell activation and enhance IgE synthesis (4, 51). This dynamic balance between membrane and soluble CD23 is considered a critical determinant of IgE homeostasis.

Beyond its role in B cells, CD23 expression has also been identified on follicular dendritic cells and, more recently, on anti-inflammatory M2 macrophages (1). Notably, CD23 + macrophages have been shown to promote expansion of regulatory T cells, highlighting an additional pathway through which CD23 may contribute to suppression of inflammatory responses (26).

Regarding its localization, CD23 expression has been confirmed on highly purified B cells. Furthermore, clinical evaluations show that in children with asthma, the proportion of B cells expressing CD23 (alongside CD21) is significantly higher compared to healthy controls (27).

CD23, primarily known as the low-affinity receptor for IgE, is notably expressed on follicular dendritic cells. Within lymphoid tissues, immunohistochemical analysis demonstrates that CD23 intensely decorates these dendritic reticulum cells. Furthermore, in specific pathological conditions such as follicular lymphoma, a characteristic tight meshwork of CD23-positive (alongside CD21-positive) cells can be observed, which represents the underlying network of follicular dendritic cells within the neoplastic follicles.

More recent publications, such as a 2021 study, provide additional insights into the regulatory role of CD23 by highlighting its high expression on anti-inflammatory M2 macrophages. It was shown that exposure to alemtuzumab (a treatment used in multiple sclerosis) induces the conversion of monocytes into anti-inflammatory macrophages with significantly upregulated CD23 expression (26). These CD23 + macrophages subsequently promote the enhancement of T-regulatory cells (specifically the CD4 + CD39 + subpopulation), demonstrating another CD23-dependent pathway that suppresses inflammatory processes.

#### Role of CD23 in fungal infection models

Experimental mouse models confirm the importance of CD23 in IgE homeostasis and pathogen-specific immunity. CD23-deficient mice display markedly elevated serum IgE concentrations, whereas transgenic overexpression of CD23 suppresses IgE-dependent humoral responses. Moreover, CD23 functions as a pattern recognition receptor (CLR, CLEC4J). In fungal infection models, CD23-deficient mice show increased susceptibility to *Candida albicans* and *Aspergillus fumigatus*, associated with impaired NF-κB signaling and reduced nitric oxide production in macrophages (28).

Beyond its role as an IgE receptor, CD23 has also been identified as a non-classical pattern-recognition receptor. Although the human form of CD23 has largely lost direct carbohydrate-binding activity during evolution, murine and bovine CD23 retain the ability to recognize fungal cell-wall polysaccharides, including mannan from *Candida albicans* and β-glucan from *Aspergillus fumigatus* (52). Additional studies suggest involvement of CD23-dependent pathways in host defense against *Cryptococcus* species, supporting a broader role for CD23 in antifungal immunity beyond IgE regulation (51).

#### Additional immune functions

Beyond its role in IgE regulation, CD23 exhibits broader immunological functions. It promotes proliferation of both normal and malignant B cells (1), displays antimicrobial activity as a C-type lectin receptor, and binds sialylated IgG, suggesting a potential role as a non-classical Fcγ receptor. CD23 signaling induces pro-inflammatory cytokine release from monocytes and dendritic cells and regulates chemokine secretion by epithelial cells, contributing to immune cell recruitment and tissue inflammation (1, 4, 5, 29). *In vivo* models further indicate that CD23 participates in shaping host responses to parasitic and viral infections, although the underlying mechanisms are not yet fully elucidated.

#### Interaction with FcεRI and effector regulation

**Figure 1:**
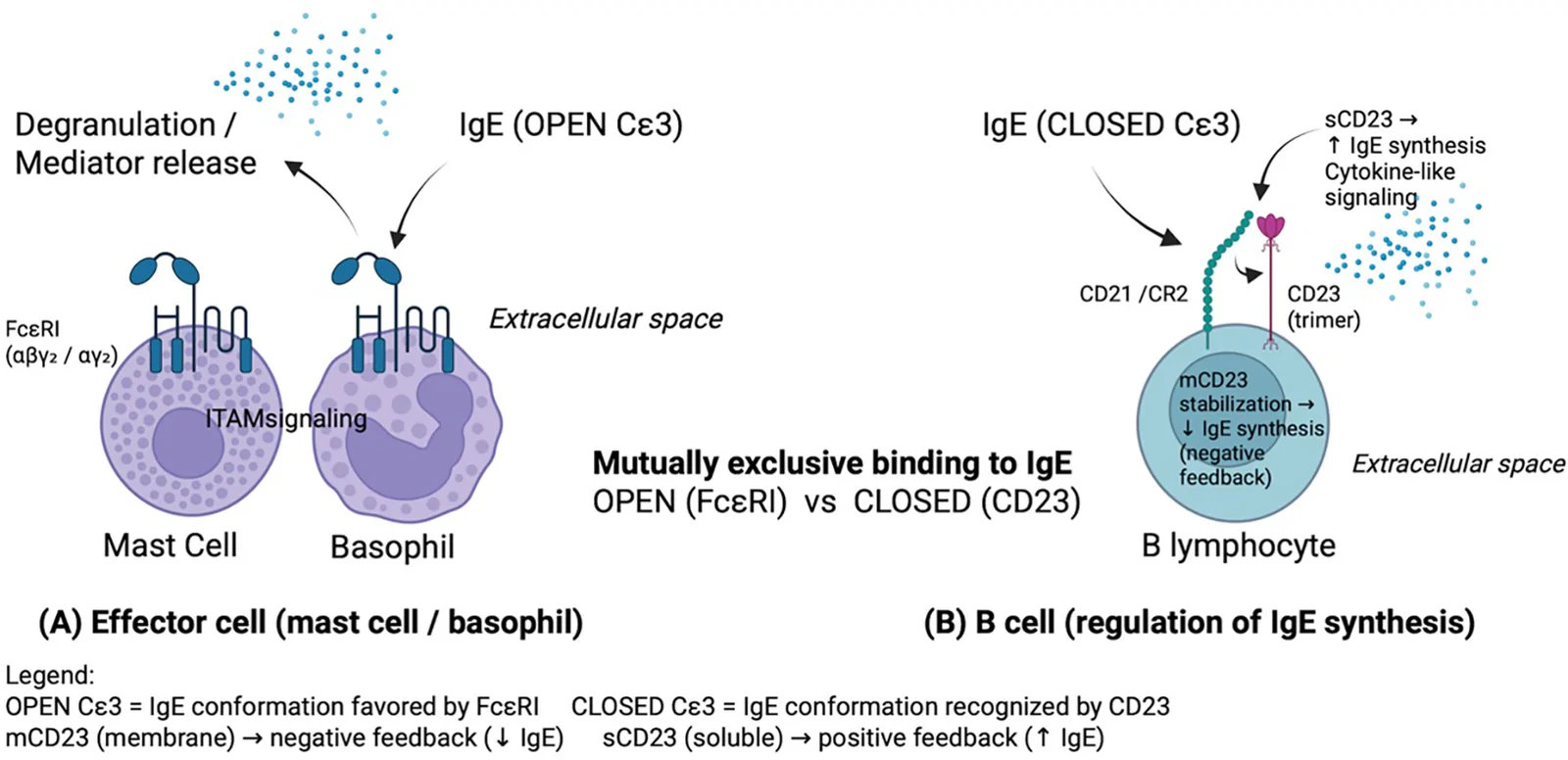
Schematic representation of the mutually exclusive binding of IgE to FcεRI (open Cε3) vs. CD23 (closed Cε3) and the CD23–CD21 feedback loop on B cells. **(A)** FcεRI engagement on effector cells drives degranulation, whereas membrane CD23 (mCD23) stabilizes negative feedback on IgE synthesis; **(B)** soluble CD23 (sCD23) promotes positive feedback and cytokine–like signaling. Own work using BioRender software.

Binding of IgE to FcεRI and CD23 is mutually exclusive due to conformational constraints within the IgE Cε3 domain. FcεRI preferentially recognizes the open conformation of IgE, whereas CD23 binds the closed conformation, preventing simultaneous engagement. Through sequestration of IgE in the closed conformation, CD23 can indirectly limit FcεRI-mediated mast cell and basophil activation, thereby modulating effector responses. This mechanism is schematically illustrated in Figure 1 (3).

Importantly, allergen-bound IgE exhibits reduced affinity for FcεRI while maintaining efficient interaction with CD23. This preferential binding may facilitate non-inflammatory clearance of IgE–allergen complexes and limit excessive mast-cell activation, thereby serving as a protective mechanism against severe systemic allergic reactions (4). These observations further support the concept that CD23 functions not only as an enhancer of immune responses but also as an important regulator preventing uncontrolled IgE-mediated effector activation.

#### Therapeutic modulation

Early therapeutic approaches targeting CD23, such as the anti-CD23 monoclonal antibody lumiliximab, reduced Th2 responses *in vitro* but failed to demonstrate clinical efficacy in asthma (1). More recent strategies using designed ankyrin repeat proteins (DARPins) have shown greater promise in preclinical studies. These agents stabilize membrane-bound CD23, reduce proteolytic shedding and release of soluble CD23, and selectively suppress IgE synthesis without affecting IgG production, supporting CD23 as a potential therapeutic target within the IgE network (13).

### Clinical evaluation and relevance of CD23 receptors in patients

#### Antigen presentation

CD23 plays a central role in IgE-facilitated antigen presentation (FAP) by binding IgE–antigen complexes and delivering them to antigen-presenting cells, thereby enhancing activation of allergen-specific T cells (6). This mechanism is particularly relevant in atopic disease, where CD23 surface density on B cells correlates with total serum IgE levels and clinical reactivity (6). Clinical studies have demonstrated that higher CD23 expression is associated with increased wheal size in skin prick testing and greater severity of cutaneous allergic symptoms.

Several clinical studies further demonstrated that CD23 surface density on B lymphocytes correlates with both total and allergen-specific IgE levels in sensitized individuals. Increased frequencies of memory B cells overexpressing CD23 have been reported in patients with asthma and atopic dermatitis, suggesting that CD23 expression reflects ongoing type 2 immune activation and may contribute to maintenance of allergic memory responses (6, 54, 55).

#### Role in parasitic and fungal immunity

CD23 contributes to protective immunity against parasitic reinfection. In *Schistosoma mansoni* infection, prospective cohort studies in Kenyan children demonstrated that higher levels of schistosome-specific IgE antibodies and an increased proportion of CD23-positive B cells were associated with greater resistance to reinfection (30). In leishmaniasis, IgE-containing immune complexes engage CD23 on macrophages, triggering nitric oxide production and facilitating parasite clearance, highlighting a direct effector role for CD23 in protozoan immunity (29).

## Soluble forms (sCD23)

Soluble isoforms of CD23 (sCD23) arise from proteolytic cleavage of membrane-bound CD23, primarily mediated by the metalloprotease ADAM10, as well as by exogenous proteases such as the house dust mite allergen Der p 1 (1, 2, 4, 5, 16). The resulting fragments vary in molecular weight but uniformly retain IgE-binding capacity and exhibit cytokine-like biological activity (1, 2, 4, 5). Circulating soluble CD23 functions as a positive regulator of IgE synthesis (1, 2, 4, 5, 25). By co-ligating complement receptor 2 (CD21) and membrane-bound IgE on B cells, soluble CD23 promotes B-cell survival, differentiation, and immunoglobulin class-switch recombination toward IgE. Beyond its effects on B cells, soluble CD23 directly modulates innate immune responses. Interaction of soluble CD23 with integrins on monocytes and macrophages induces nitric oxide production and activates intracellular signaling pathways, including MAPK, ERK, and NF-κB, leading to the release of pro-inflammatory cytokines (1, 5, 28, 29). Through these mechanisms, soluble CD23 acts as a cytokine-like mediator that amplifies and sustains inflammatory responses.

Soluble CD23 is increasingly recognized as a clinically relevant biomarker of chronic immune activation. Elevated circulating concentrations have been reported not only in allergic disorders but also in conditions such as severe malaria and heart failure with reduced ejection fraction, where serum levels correlate with disease severity and progression (51, 53). These observations support the concept that sCD23 reflects broader immune activation beyond classical allergic disease.

### Experimental regulation of IgE homeostasis in *in vivo* models

Experimental models further confirm the regulatory role of CD23 and its soluble forms in IgE homeostasis. CD23-deficient mice exhibit enhanced IgE responses, whereas transgenic overexpression of CD23 suppresses IgE production (24). Importantly, strategies that stabilize membrane-bound CD23 reduce proteolytic cleavage, decrease soluble CD23 release, and attenuate IgE synthesis (13). These findings provide a mechanistic rationale for targeting CD23 shedding, through ADAM10 inhibition or CD23-directed biologics, as potential therapeutic interventions within the IgE network.

### Clinical evaluation and correlation with clinical disease activity indices

Clinical studies consistently demonstrate elevated soluble CD23 levels in atopic dermatitis, chronic spontaneous urticaria, and particularly bullous pemphigoid (4, 7, 8, 11, 12, 21, 33–35). In these conditions, soluble CD23 concentrations in serum and, in the case of bullous pemphigoid, blister fluid correlate with total IgE levels and validated disease activity indices. Reported correlations typically range from *r* ≈ 0.5–0.7 between soluble CD23 and clinical scores such as EASI, UAS7, and BPDAI (6) supporting the potential utility of soluble CD23 as a biomarker of disease activity and therapeutic response (7, 8).

Additional evidence comes from studies in atopic dermatitis, where reductions in CD23 expression on B lymphocytes following selective IgE apheresis closely paralleled improvements in SCORAD scores (57). Similarly, correlations between CD23 expression and sensitization profiles against specific molecular allergens have been reported, suggesting that CD23 may serve not only as a marker of disease activity but also as an indicator of allergen-specific immune responses (58).

In healthy individuals, baseline serum soluble CD23 concentrations typically range from approximately 5–15 ng/mL (5, 7, 8). In contrast, patients with IgE-driven inflammatory diseases, including atopic dermatitis and bullous pemphigoid, frequently exhibit significantly elevated levels, commonly in the range of 30–80 ng/mL (7, 8), correlating with total serum IgE concentrations and clinical disease activity. In chronic lymphocytic leukemia, serum soluble CD23 levels may exceed 100 ng/mL and are used as a prognostic marker of disease burden and progression.

## FcεRII/CD23 in skin disorders

### Allergic skin diseases

#### Atopic dermatitis

In clinical cohorts presenting with atopic dermatitis, serum levels of soluble CD23 are markedly elevated (44, 46), with approximately 60%–70% of active cases exhibiting values above the normal range. Increased soluble CD23 concentrations correlate with total serum IgE levels and disease severity, with reported correlation coefficients ranging from *r* ≈ 0.4–0.6 for EASI scores. These findings support the potential role of soluble CD23 as a biomarker of disease activity in atopic dermatitis. In addition, CD23 expressed on B cells enables IgE-facilitated allergen presentation, thereby enhancing T-cell activation. High CD23 surface density on naïve B cells correlates with both total IgE levels and the magnitude of skin test reactions in atopic individuals (6).

Recent studies have demonstrated that CD23 expression on B lymphocytes is strongly associated with sensitization to specific molecular allergens, including *Cladosporium herbarum*, Fel d 1, and Bla g 9 (57). Interestingly, dupilumab therapy appears to strengthen this association, raising the possibility that IL-4Rα blockade may enhance CD23-mediated non-inflammatory clearance of IgE-containing immune complexes (57). These findings further support a functional role for CD23 in regulating allergen-specific immune responses in atopic dermatitis.

#### Food allergy

In cohorts sensitized to peanut, hazelnut, or egg allergens, reported serum soluble CD23 levels have been inconsistent, possibly reflecting variability in soluble isoforms or differences in assay methodology. Median concentrations are generally slightly higher (20–30 ng/mL) in food-allergic patients compared with healthy controls (10–15 ng/mL), although substantial overlap limits diagnostic utility. By contrast, soluble FcεRI has demonstrated stronger and more reproducible associations with clinical tolerance. Accordingly, the precise role of soluble CD23 as a biomarker in food allergy remains uncertain (31). Beyond soluble biomarkers, recent studies identified a population of IgG1 + IL4R + CD23 + memory B cells that may serve as precursors of high-rate IgE-producing cells in food allergy, particularly in peanut allergy (54, 55). These findings suggest that CD23 may contribute to maintenance of long-term allergic memory and rapid reactivation of IgE responses following allergen exposure. For example, Steinert et al. reported median soluble CD23 concentrations of 43.03 (57.95) U/mL in food-allergic patients compared with 94.68 (107) U/mL in healthy controls, underscoring the heterogeneity of available data (31).

#### Chronic spontaneous urticaria

IgE plays a central role in the autoallergic type I endotype of chronic spontaneous urticaria, whereas IgG autoantibodies predominate in autoimmune type IIb disease (11, 12, 43). CD23 may participate in IgE regulation and antigen presentation primarily within the type I endotype. Preliminary studies indicate that serum soluble CD23 levels are modestly elevated in CSU compared with healthy controls, with mean values of approximately 20–40 vs. 10–15 ng/mL, and may correlate with total IgE concentrations (33, 35). Small cohort studies have suggested that higher baseline soluble CD23 levels could predict a better clinical response to omalizumab, although findings remain inconsistent and have not been validated in prospective trials. Emerging biomarker panels incorporating total IgE, anti-thyroid peroxidase IgG antibodies (32), and basophil counts indicate that CD23 expression and soluble levels may contribute to patient stratification, but robust clinical evidence is still lacking (11, 12, 33–35).

Emerging anti-IgE therapies may also indirectly affect CD23-dependent pathways. The anti-IgE monoclonal antibody UB-221 has been shown to suppress *de novo* IgE synthesis while preserving formation of stable IgE–CD23 complexes on B cells, thereby enhancing physiological CD23-mediated inhibitory signaling (59). This mechanism differs from that of omalizumab and may represent a novel strategy for modulating the IgE–CD23 axis in chronic spontaneous urticaria.

Structural studies demonstrating mutually exclusive binding of IgE to FcεRI and CD23 support the concept of a regulatory switch between effector activation and immune modulation. From a therapeutic perspective, blocking CD23–IgE interactions may reduce allergen presentation and downstream T-cell activation, thereby complementing anti-IgE strategies such as omalizumab and ligelizumab. Experimental data support this concept, as lumiliximab reduced Th2 responses and IgE production *in vitro*, while DARPins targeting CD23 stabilized its membrane-bound form and limited soluble CD23 release. In addition, omalizumab therapy in allergic asthma has been associated with reduced CD23 expression on B cells, suggesting indirect modulation of the CD23–IgE axis (3, 6, 13).

### Autoimmune blistering diseases

#### Bullous pemphigoid

Bullous pemphigoid is increasingly recognized as an IgE-associated autoimmune disease. Up to 70%–85% of patients exhibit elevated circulating IgE levels, most commonly directed against the NC16A domain of BP180 and, less frequently, BP230. Both FcεRI and CD23 are implicated in amplifying these IgE-driven immune responses (9, 10).

Serum and blister-fluid levels of soluble CD23 are significantly higher in patients with bullous pemphigoid than in healthy controls and correlate with disease severity and total IgE concentrations (7, 8). Reported mean serum soluble CD23 levels range from approximately 35–60 ng/mL in BP patients compared with 10–15 ng/mL in controls, with positive correlations to BPDAI scores (*r* ≈ 0.5–0.7, *p* < 0.01). These findings suggest that the IgE–CD23 axis contributes to disease pathogenesis. Mechanistically, CD23 expression on B cells and potentially keratinocytes may enable IgE-facilitated antigen presentation and promote Th2-skewed immune responses, while elevated soluble CD23 reflects ongoing proteolytic cleavage and active immune signaling.

Clinical reports and small case series indicate potential benefit of anti-IgE therapy with omalizumab in refractory bullous pemphigoid, including reductions in FcεRI expression, circulating IgE levels, and lesional eosinophilia despite persistence of IgG autoantibodies (9, 10, 36). These observations suggest that patients with a predominant IgE/CD23 profile may be particularly responsive to anti-IgE biologics. However, no randomized controlled trials have been completed, and existing evidence is limited to small observational cohorts, underscoring the need for larger prospective studies. Overall, CD23 emerges in bullous pemphigoid as both a biomarker, reflected by elevated soluble CD23 in serum and blister fluid, and a potential therapeutic target, although prospective validation is still required.

#### Parasitic and infectious skin diseases

Direct dermatologic data on CD23 in parasitic and infectious skin diseases are limited; however, immunological studies suggest a role for CD23 in antiparasitic immunity, particularly against helminths (1, 2, 30, 40). Soluble CD23 may enhance IgE production and eosinophil recruitment, mechanisms relevant to parasite-associated dermatoses such as cutaneous larva migrans. Experimental studies in schistosomiasis and onchocerciasis models demonstrate upregulation of CD23 on B cells and monocytes, correlating with IgE-mediated protective responses and eosinophilia.

In *Plasmodium falciparum* infection, surface expression of CD23 on B lymphocytes is increased through direct interaction with the CIDR1α domain of PfEMP1, accompanied by upregulation of activation markers including HLA-DR, CD40, CD80, and CD86, and associated with B-cell proliferation and immunoglobulin production (42). In schistosomiasis caused by *Schistosoma mansoni*, elevated CD23b mRNA expression in B lymphocytes reflects activation that supports antibody production but may also contribute to chronic inflammation (39). In contrast, *Schistosoma haematobium* infection displays a paradoxical pattern in which circulating soluble CD23 levels increase with infection intensity but decline with higher parasite-specific IgE levels, suggesting an immunoregulatory role for soluble CD23 in balancing protective immunity and immune regulation (40).

In onchocerciasis, transcriptomic analyses revealed no differential CD23 expression compared with non-endemic controls, indicating that CD23 regulation may occur post-transcriptionally or play a limited role in systemic immune responses. Data on CD23 expression in filariasis caused by *Wuchereria bancrofti* are lacking, representing a notable gap in the literature.

Indirect evidence also links CD23 to viral modulation. Epstein–Barr virus induces CD23b expression on B cells via latent membrane protein 1–mediated signaling, thereby amplifying IgE responses and altering B-cell survival (17). In dermatology, this raises the hypothesis that CD23 may contribute to EBV-associated cutaneous lymphoproliferative disorders, although direct evidence remains limited.

#### Cutaneous malignancies

CD23 is a recognized marker of B-cell activation and has established diagnostic and prognostic relevance in chronic lymphocytic leukemia, mantle cell lymphoma, and plasmacytomas. In dermatology, cutaneous B-cell lymphomas, including primary cutaneous follicle center lymphoma and marginal zone lymphoma, may exhibit aberrant CD23 expression, supporting its use in immunophenotypic diagnostics (1). However, systematic studies addressing the prevalence, intensity, and prognostic significance of CD23 expression in primary cutaneous lymphomas are scarce, and current knowledge largely relies on extrapolation from nodal hematologic malignancies. Elevated serum soluble CD23 levels correlate with tumor burden in CLL (47), suggesting potential extension of CD23 monitoring to cutaneous lymphomas, although available data remain preliminary.

An overview of dermatological conditions associated with altered CD23 and soluble CD23 expression is summarized in Table 3.

## Therapeutic potential

**Figure 2:**
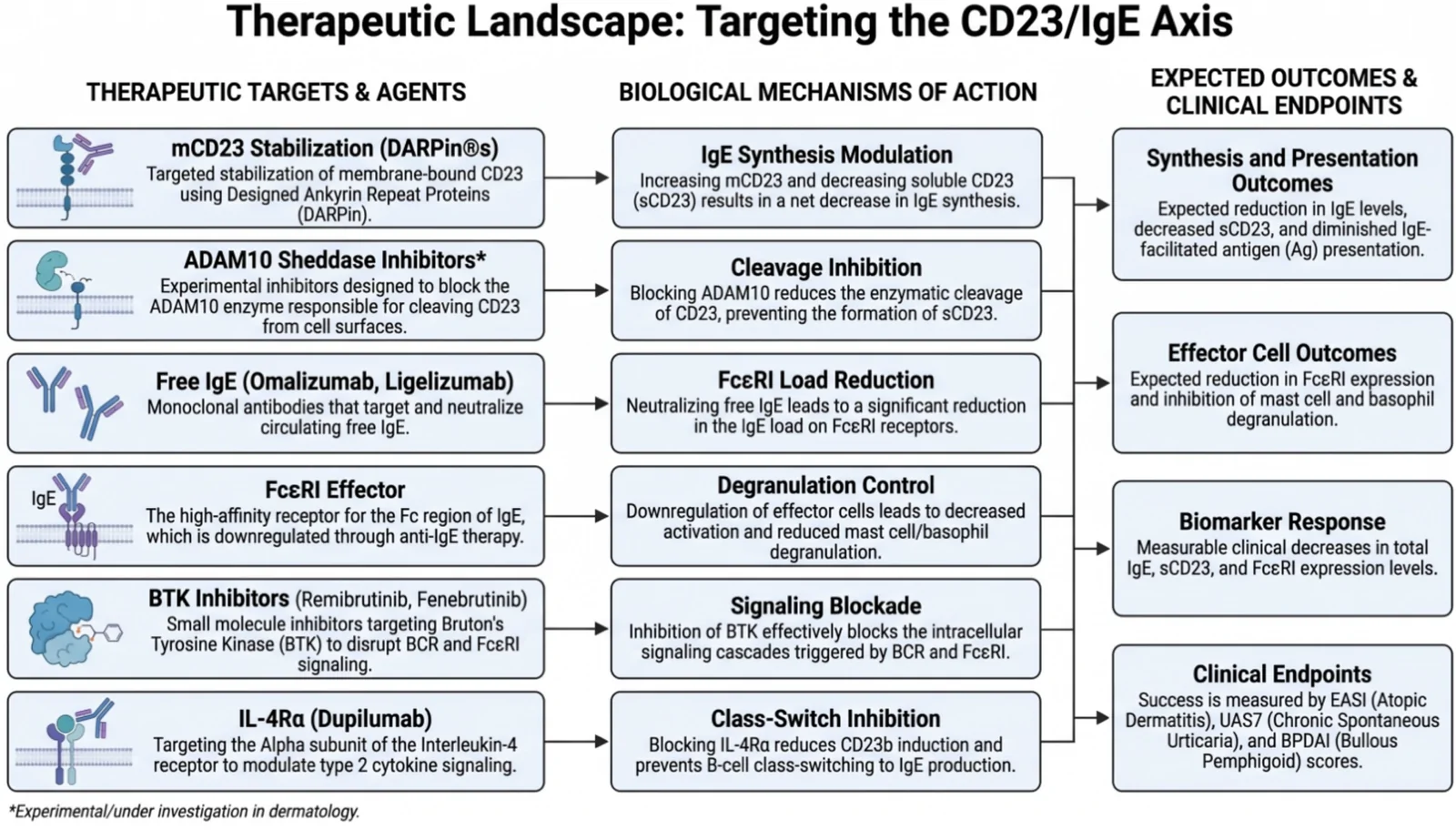
Therapeutic Landscape Targeting the CD23/IgE Axis.

The dual and context-dependent nature of FcεRII/CD23, acting as both a negative regulator of IgE synthesis and a potential amplifier of inflammatory signaling through its soluble forms, creates multiple therapeutic entry points within the IgE network. Potential strategies include stabilization of membrane CD23, inhibition of proteolytic cleavage, blockade of IgE binding, and modulation of upstream or downstream signaling pathways that converge on IgE-driven inflammation (1–5, 13). A conceptual overview of druggable nodes within the CD23/IgE axis, together with their principal mechanisms of action and anticipated biomarker and clinical outcomes, is summarized in Figure 2.

### Limitations and incomplete *in vivo* efficacy of direct CD23 blockade

Direct CD23 blockade has demonstrated biological activity in experimental models; however, its *in vivo* efficacy appears to be incomplete (1, 13, 38, 41). This reflects the multifunctional nature of CD23, which is involved not only in the regulation of IgE synthesis, but also in allergen-IgE immune complex handling, antigen presentation, epithelial transcytosis, and modulation of B-cell signaling. Blockade of CD23 does not completely arrest the IgE network, as partial or incomplete saturation of CD23 fails to establish a functional allosteric switch *in vivo*. Consequently, most of therapeutic strategies aimed at modulating the IgE network via CD23 structural manipulation remain confined to preclinical models. While *in vivo* knockout and transgenic models confirm that restricting proteolytic cleavage and sCD23 release effectively suppresses IgE-mediated humoral responses, these interventions require rigorous clinical trial validation (13, 24). Although the impact of environmental and microbial factors on receptor cleavage and downstream signaling is well-characterized *in vitro* and in cellular models, directly targeting these pathways to restore IgE homeostasis in humans remains a key focus of ongoing preclinical development. Direct CD23 blockade can reduce allergic inflammation in experimental models, but its *in vivo* efficacy appears incomplete. CD23 functions as a pleiotropic and partially redundant regulator rather than a single dominant effector pathway so CD23-blocking therapies may be more effective as part of broader combinational approaches than as stand-alone treatments (15).

### Direct modulation of CD23

Designed ankyrin repeat proteins (DARPins) with picomolar affinity for CD23 represent a highly selective approach to modulating IgE synthesis. These molecules stabilize membrane-bound CD23, reduce proteolytic shedding, and selectively suppress IgE production at a post-class-switch level without affecting IgG synthesis. Among these agents, the bispecific DARPin D89-86 demonstrated the strongest effects, increasing CD23 surface density and reducing mature IgE transcripts in preclinical models (13). To date, no clinical studies have evaluated DARPin-based CD23 targeting in dermatologic diseases. Proof-of-concept trials in atopic dermatitis, chronic spontaneous urticaria, or bullous pemphigoid, using endpoints such as EASI, UAS7, and BPDAI together with biomarker assessment, represent a logical next step.

Additional experimental approaches include monoclonal antibodies and engineered protein scaffolds capable of selectively disrupting CD23–IgE interactions while preserving other immune functions. These molecules reduce allergen-driven inflammatory signaling and may offer greater selectivity than conventional anti-IgE therapies (59, 60).

Anti-CD23 monoclonal antibodies, such as lumiliximab, reduced Th2 responses and IgE production *in vitro*; however, clinical trials in asthma failed to demonstrate meaningful efficacy (1). No dermatologic studies have been conducted. Compared with DARPins, monoclonal antibodies act through classical Fc-mediated effector mechanisms and have longer systemic persistence, which may increase the risk of off-target immunosuppression. In contrast, DARPins are smaller, non-immunoglobulin scaffolds characterized by rapid tissue penetration, shorter half-life, and allosteric stabilization of membrane CD23 rather than Fc-dependent depletion, potentially allowing for more selective modulation of IgE biology.

### Inhibition of CD23 proteolysis

ADAM10 is the principal sheddase responsible for CD23 cleavage, and its inhibition reduces soluble CD23 release and IgE production in experimental models (1, 2, 4, 5). However, ADAM10 also cleaves multiple substrates, including Notch receptors, E-cadherin, and cytokine receptors, raising substantial safety concerns for systemic inhibition. Future strategies may therefore require selective or locally delivered approaches, such as topical ADAM10 inhibitors, to reduce soluble CD23 while minimizing off-target effects.

### Blockade of CD23-mediated transcytosis

In airway models, anti-CD23 antibodies inhibited IgE–antigen transcytosis across epithelial barriers and reduced eosinophilic inflammation and airway hyperreactivity (38). As these data are currently restricted to respiratory models, *ex vivo* studies using human skin are needed to determine whether similar CD23-dependent transport mechanisms operate in the cutaneous barrier.

Evidence from epithelial cell models demonstrates that RNA interference-mediated suppression of CD23 expression almost completely abolishes bidirectional IgE transcytosis (49). These findings support epithelial CD23 as a potential therapeutic target for reducing allergen penetration and amplification of local allergic inflammation.

### Anti-IgE biologics and indirect effects on CD23

Anti-IgE therapies such as omalizumab and ligelizumab neutralize free IgE, destabilize FcεRI complexes, and indirectly reduce CD23-dependent antigen presentation (3, 14). In chronic spontaneous urticaria, omalizumab is the standard of care for antihistamine-refractory disease, achieving complete response in approximately 35%–40% of patients and partial response in an additional ∼50%. The IgG anti-thyroid peroxidase to total IgE ratio has been proposed as a predictor of poor therapeutic response (33).

In bullous pemphigoid, case reports and small observational cohorts comprising approximately 20–30 patients have reported clinical improvement with omalizumab, accompanied by reduced FcεRI expression, decreased circulating IgE, and lower lesional eosinophil counts despite persistence of IgG autoantibodies (36).

Soluble IgE receptors such as sFcεRI may also contribute to IgE regulation by capturing free IgE and preventing mast cell activation. Although clinical data link sFcεRI to tolerance in food allergy, its role in bullous pemphigoid and chronic spontaneous urticaria has not been investigated (31). Parallel assessment of soluble CD23 and sFcεRI in these diseases, correlated with response to anti-IgE therapy, may clarify their complementary biomarker potential.

### Targeting adjacent pathways within the IgE network

Interleukin-4 and interleukin-13 upregulate the CD23b isoform, linking type 2 cytokine signaling to IgE amplification (1, 2, 4, 25). Blockade of IL-4Rα with dupilumab may therefore indirectly reduce CD23 signaling and IgE production (4, 25). However, data on changes in soluble CD23 levels during dupilumab therapy are currently lacking, representing an important research gap.

Bruton's tyrosine kinase inhibitors, including remibrutinib and fenebrutinib, target signaling downstream of both FcεRI and the B-cell receptor (37). New-generation BTK inhibitors demonstrate rapid and sustained efficacy in chronic spontaneous urticaria, with phase 3 trials ongoing. Although not directly targeting CD23, these agents modulate the IgE network and may influence CD23–IgE crosstalk. Future trials should incorporate soluble CD23 measurements as mechanistic biomarkers to assess indirect effects on CD23 activity.

### Biomarkers and patient stratification

Candidate biomarkers within the CD23/IgE axis include soluble CD23, measured in serum and blister fluid, which correlates with disease activity in bullous pemphigoid, atopic dermatitis, and chronic spontaneous urticaria (4, 7, 8, 11, 12, 21, 33–35). CD23 surface density on B cells is associated with total IgE levels and IgE-facilitated antigen presentation (6). Additional stratification markers include total IgE, eosinophilia, and the IgG anti-thyroid peroxidase to IgE ratio in chronic spontaneous urticaria (6).

In chronic spontaneous urticaria, two major endotypes are recognized: type I (autoallergic, IgE-driven) and type IIb (autoimmune, IgG-driven), with CD23 being more relevant in the type I endotype (11, 12). In bullous pemphigoid, a phenotype characterized by high IgE levels, eosinophilia, and elevated soluble CD23 may identify patients most likely to benefit from anti-IgE therapy (7–10, 36). These observations support the development of endotype- and biomarker-guided therapeutic algorithms.

Beyond soluble CD23 measurements, expansion of type 2 memory B-cell populations characterized by the IgG + IL4R + CD23 + phenotype may provide an additional biomarker of enhanced IgE-producing potential (54, 55). Such cellular biomarkers could complement serum-based markers and improve identification of patients most likely to benefit from therapies targeting the IgE–CD23 axis.

**Figure 3:**
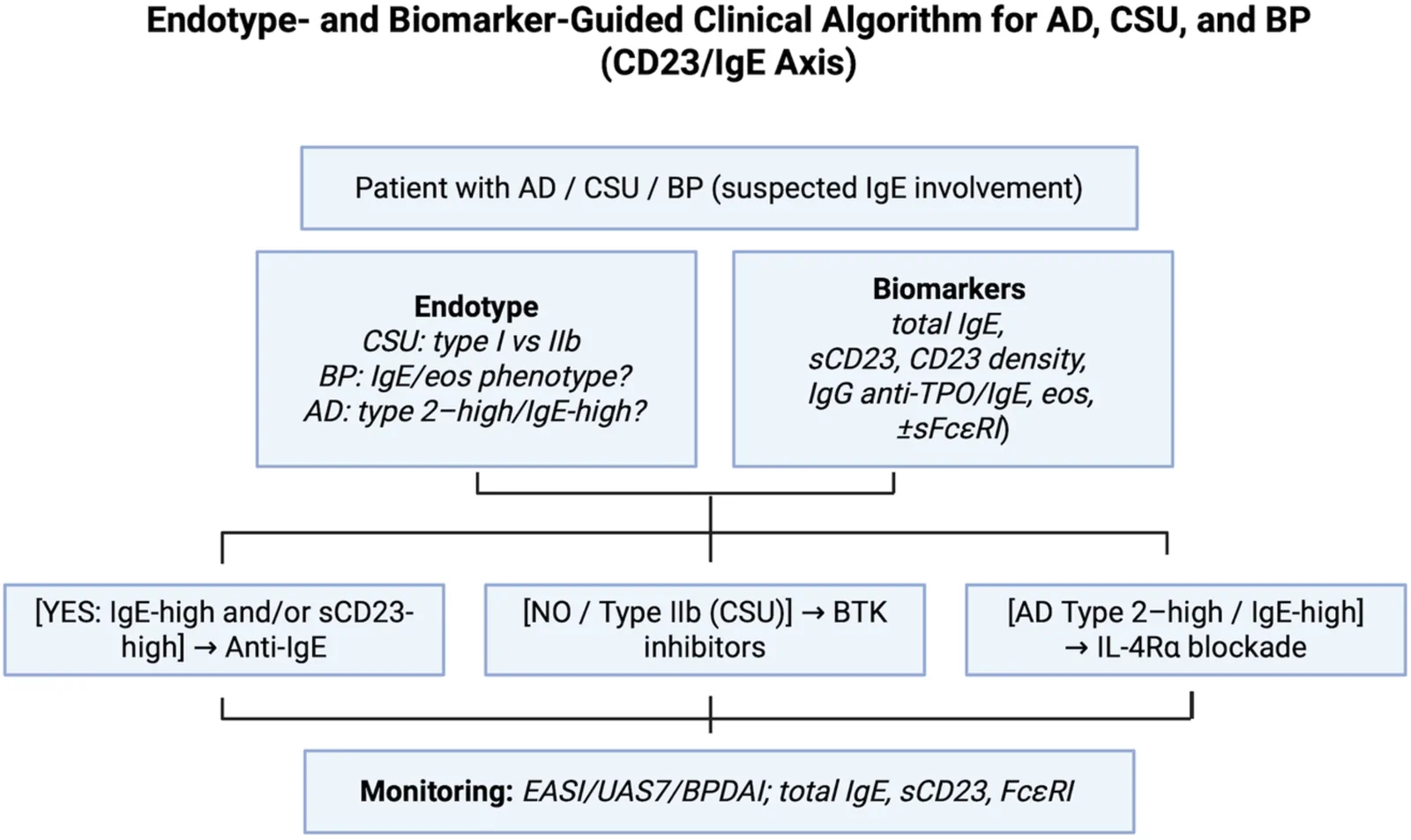
Endotype- and Biomarker-Guided Clinical Algorithm for AD, CSU, and BP (CD23/IgE Axis).

A stepwise clinical workflow integrating disease endotypes, biomarker profiles, and therapeutic decision-making is outlined in Figure 3.

### Safety considerations

Safety considerations for CD23-targeted and related therapeutic strategies include potential off-target toxicity associated with ADAM10 inhibition due to its broad substrate spectrum, as well as the need to monitor for hypersensitivity reactions and modulation of humoral immunity with anti-CD23 and anti-IgE therapies. New-generation BTK inhibitors demonstrate improved cardiovascular and bleeding safety profiles compared with first-generation agents, although careful monitoring remains warranted (37). Key adverse events, monitoring requirements, and the current level of evidence for CD23-targeted and adjacent therapeutic strategies are summarized in Table 4.

**Table 4:** Safety considerations of CD23-targeted and adjacent therapeutic strategies.

Drug class/Target	Examples	Key adverse events	Monitoring/Notes	Evidence
Anti-CD23/CD23modulators	Lumiliximab; anti-CD23 DARPins (e.g., D89-86)	Infusion/hypersensitivity reactions; potential effects on B-cell function (theoretical)	Limited clinical data; DARPins: selective IgE suppression without Fcγ engagement (preclinical)	(Acharya, 2010; Fellmann, 2015)
Anti-IgE	Omalizumab; Ligelizumab	Injection-site reactions; rare anaphylaxis; serumsickness–like; headache	Standard in CSU; may lower FcεRI expression; consider IgGanti-TPO/IgE ratio as poorresponse marker	(Kolkhir, 2022; Brás, 2023; McDonnell, 2023)
ADAM10 inhibitors	Experimental small-molecule/biologic inhibitors	Off-target toxicity (Notch/cadherins/cytokines); possible barrier effects	Preclinical rationale to ↓sCD23; consider topical/selective strategies	(Acharya, 2010; Conrad, 2007)
BTK inhibitors	Remibrutinib; Fenebrutinib	Headache, diarrhea, infections; bleeding risk; older BTK-i: arrhythmias	Rapid onset in CSU trials; newer agents with improved safety vs. firstgen	(Bernstein, 2024)
IL-4Rα blockade	Dupilumab	Conjunctivitis, transient eosinophilia, injection-site reactions	Robust longterm safety in AD; indirectly ↓CD23b/IgE axis	(Engeroff, 2021)

### Summary of therapeutic implications

FcεRII/CD23 represents a key regulatory checkpoint within IgE biology. Stabilization of membrane CD23 or reduction of soluble CD23 release has the potential to attenuate IgE synthesis and downstream inflammatory cascades (13, 24). Anti-IgE biologics are already validated in chronic spontaneous urticaria and show promise in bullous pemphigoid, while CD23-related biomarkers may refine patient selection and treatment stratification (9–12, 33–36). Modulation of adjacent pathways, including IL-4Rα and BTK signaling, offers additional opportunities for synergistic intervention within the IgE network (4, 25, 37).

## Future directions and research gaps

Despite growing evidence supporting a role for FcεRII/CD23 in dermatologic diseases, several key questions remain unresolved, limiting its current clinical applicability.

### Biomarker validation

Although serum soluble CD23 levels correlate with total IgE concentrations and disease activity in atopic dermatitis (4, 21) and bullous pemphigoid (7, 8), robust clinical validation is still lacking. Large, prospective, multicenter studies are required to determine its sensitivity, specificity, and predictive value across different disease stages and treatment modalities. Importantly, the absence of standardized assays and clinically meaningful cut-off values currently represents a major barrier to translation into routine practice.

### Endotype stratification

Emerging data suggest that CD23 may contribute to distinguishing IgE-driven (autoallergic) from autoimmune (type IIb) endotypes in chronic spontaneous urticaria (11, 12, 33–35), as well as IgE/eosinophilic vs. non-IgE phenotypes in bullous pemphigoid (9, 10, 36). However, prospective studies linking CD23-related biomarker profiles to therapeutic responses and long-term outcomes are still missing.

### Therapeutic targeting

Direct CD23-targeted therapies remain experimental. Although anti-CD23 DARPins and monoclonal antibodies effectively reduce IgE synthesis in preclinical models (13), no dermatology-specific clinical trials have been conducted to date. Inhibition of CD23 proteolysis via ADAM10 blockade is mechanistically attractive but raises significant safety concerns due to the broad substrate spectrum of this protease (1, 2, 4, 5). Development of selective or locally delivered approaches may be required before clinical application can be considered.

### Systems immunology and integrative approaches

Future studies integrating CD23 into broader IgE-related signaling networks, including FcεRI, BTK, and IL-4Rα pathways, may provide a more comprehensive understanding of type 2 inflammation in the skin (3, 4, 25, 37). Systems immunology approaches, combined with computational modeling and longitudinal biomarker profiling, could clarify how modulation of CD23 influences immune balance and therapeutic response.

### Additional research gaps

Key unmet needs include prospective validation of soluble CD23 as a biomarker in atopic dermatitis, chronic spontaneous urticaria, and bullous pemphigoid; development and harmonization of standardized laboratory assays; and mechanistic studies dissecting the CD23–CD21 feedback loop in cutaneous autoimmunity. Moreover, potential differences in CD23 biology between pediatric and adult populations remain largely unexplored and warrant systematic investigation.

## Conclusions

FcεRII (CD23) is increasingly recognized as a central regulatory node within the IgE network, integrating immune regulation and effector pathways that are directly relevant to inflammatory skin disease (1, 2, 4, 5). Evidence from atopic dermatitis (4, 21), chronic spontaneous urticaria (11, 12, 33–35), and bullous pemphigoid (7–10, 36) indicates that both membrane-bound and soluble forms of CD23 contribute to disease pathogenesis through distinct yet interconnected mechanisms that shape IgE homeostasis, antigen presentation, and downstream inflammation.

Elevated circulating levels of soluble CD23 consistently associate with increased disease activity and IgE-driven immune responses, supporting its potential role as a biomarker of disease burden and immunological activity (7, 8). In parallel, experimental strategies aimed at stabilizing membrane CD23 or limiting its proteolytic cleavage demonstrate that selective modulation of this pathway can attenuate IgE synthesis and effector signaling, providing a compelling mechanistic rationale for therapeutic exploration (13, 24).

Although CD23 has not yet reached the level of a validated clinical biomarker or drug target in dermatology, its positioning at the intersection of IgE regulation, FcεRI signaling, and type 2 inflammation makes it uniquely suited for integration into emerging treat-to-target and precision medicine frameworks. Combining CD23-related parameters with established markers such as total IgE, FcεRI expression, and eosinophilia may refine patient stratification, particularly in heterogeneous conditions such as chronic urticaria and bullous pemphigoid.

Ultimately, advancing CD23 from an immunological concept to a clinically actionable tool will require prospective validation studies, standardized assays, and disease-specific clinical trials. Nevertheless, the accumulating body of evidence positions CD23 as a mechanistically distinctive and clinically relevant element of the IgE axis, with the potential to inform both biomarker development and next-generation therapeutic strategies in dermatology.
